# Captivity and geography influence the antibiotic resistome of non-human primates

**DOI:** 10.3389/fvets.2022.1020276

**Published:** 2022-11-18

**Authors:** Hongli Huang

**Affiliations:** ^1^Clinical Biological Specimen Bank, Discipline Construction Office, The First Affiliated Hospital of Guangxi Medical University, Nanning, China; ^2^Life Sciences Institute, Guangxi Medical University, Nanning, China

**Keywords:** antibiotic resistance genes, captivity, geography, metagenome, non-human primate fecal

## Abstract

**Introduction:**

Antibiotic resistance poses a serious threat for animals and humans health worldwide. Yet a comprehensive exploration of the influence of captivity and geography on non-human primate (NPH) gut antibiotic resistance remains incomplete.

**Methods:**

In this study, 131 metagenomic sequencing datasets of five species of NHPs included different regions and lifestyles were selected to perform the antibiotic resistance analysis.

**Results:**

Nineteen related resistance antibiotics and 325 antibiotic resistance genes (ARGs) were obtained. A significantly higher abundance and diversity index of ARGs in the captive NHPs than in the wild was found but not for all of the samples. The biomarker-tracking of ARGs analysis identified key ARGs related to aminoglycoside resistance genes and tetracycline resistance genes.

**Discussion:**

These results suggest that captivity and geography changes associated with human activities can lead to marked changes in the ecology of the NHP gut flora ARGs.

## Introduction

Antibiotic resistance genes (ARGs) existed before antibiotics were reported and did not pose a threat to humans at that time. However, the widely used antibiotics in clinical, aquaculture, animal husbandry, and planting accelerated the propagation and dissemination of ARGs (1). Nowadays, antibiotic resistance has been recognized as a vital global threat to human and public health by World Health Organization (2). The World Bank warned that antibiotic resistance would cause 10 million deaths and 100 trillion dollars losses in global economic by 2050 if unchecked (3, 4). European Antimicrobial Resistance Surveillance Network (http://www.ecdc.europa.eu/en/activities/surveillance/EARS-Net) and the US National Antimicrobial Resistance Monitoring System for Enteric Bacteria (http://www.cdc.gov/narms/) focus on the clinical and public dosage of antibiotics and the isolation of drug-resistant bacteria in public health laboratories, also illustrating that antibiotic resistance has been a wide concern (5). In almost all environments, sediment (6), surface water (7, 8), sewage (9–11), sludge (12), medical waste (13), and animal feces (14–16) serve as important reservoirs of ARGs that provide for reproduction and propagation conditions. At the same time, ARGs can transmit by horizontal gene transfer (17–20) between pathogen or host (21) *via* mobile genetic elements (4, 22, 23), which will increase the difficulty of environmental governance and disease treatment. Thus, under the One-Health concept, effective measures should be researched to understand and control ARGs transmission.

Non-human primates, as close living relatives of human beings in the world, have a high degree of genetic and physiological similarity to humans (24). They are considered as the best model animal to explore the occurrence and development of human disease in medicine. However, due to the study of animals referred to ethical issues and part of the NPHs being endangered or protected animals, samples of them are hard to get. The high-throughput sequencing-based metagenomic analysis is a powerful tool that provided conveniences for rare animal samples microbiota analysis. Thus, systematic research on the resistome of NPHs by metagenomic analysis will provide us with the prevention of human diseases. Gut microbial communities, affected by several factors, including host lifestyle, host species, and geography (25), can generally shape the resistome (26, 27). Therefore, hypothesis that NHPs in wild might harbor more diverse ARGs and lower abundance than that in captivity were put forward since their surroundings were varied and they almost free from antibiotic selection pressure. Comparison of the gut resistome of NHPs in wild and in captivity can help us have a deep understanding of how the lifestyles influence the gut resistome. Besides, geography may also be an important factor that influences the ARGs abundance and diversity. To uncover these mysteries, five species of NHPs from different regions and lifestyles were included in our study.

Studies that looked at ARGs in gut microbiota of human or other animals have usually been performed at a local scale, which reduced the statistical power of the analyses. To break through this limitation, ARGs abundance and diversity of NHPs from a large scale were obtained. In this study, *Macaca mulatta* (*M. mulatta*) datasets sampled and sequenced in previous studies were included. Metagenomic datasets of NHPs worldwide including white-faced capuchins (*Cebus capucinus*), *Macaca sp, Macaca fascicularis* (*M. fascicularis*), and *Rhinopithecus roxellana* (*R. roxellana*) that held in the Sequence Read Archive (SRA, https://www.ncbi.nlm.nih.gov/sra) were download. Metagenomic data of white-faced capuchins, *Macaca sp., M. mulatta* and *M. fascicularis* could be classified by the host, lifestyle, and geography and datasets for *R. roxellana* were classified into captive, semi-wild, and wild groups. Hence, this research aimed to (1) investigate the distribution of gut resistome from large-scale NHPs; (2) analyze the biomarker ARGs of captive NHPs by Linear discriminant analysis (LDA) effect size (LEfSe) algorithm; (3) speculate the influence of captivity and geography on the abundance and diversity of NHPs gut resistome.

## Materials and methods

### Dataset collection

A total of 131 metagenome datasets were included in this study. Among them, 34 *M. mulatta* (Guangxi) were from the datasets of samples collected in the early stage sequenced by Illumina X-ten strategies, and 19 white-faced capuchins (PRJNA485217, Costa Rica), 23 *Macaca sp*. (PRJNA478556, USA, Minneapolis), 17 *M. fascicularis* (PRJEB22765, China, Beijing), and 38 *R. roxellana* (PRJNA436633, China, Hubei) were downloaded from the SRA database of National Center for Biotechnology Information (NCBI) (https://www.ncbi.nlm.nih.gov/sra/?term=) (1, 28). The sample ID, sequencing platform, reads length, reads number, data size, and accession numbers of the downloaded datasets were listed in Supplementary Table S1. The datasets of SRA format were converted to the fastq format by the fastq-dump module integrated in the NCBI SRA Toolkit (http://www.ncbi.nlm.nih.gov/Traces/sra/view5software) for subsequent analysis.

### Bioinformatics analysis

The sequencing reads were aligned to a hierarchical structured Structured Antibiotic Resistance Genes (SARG) database (containing 24 ARG types and 1209 ARG subtypes) to characterize the resistance structure by the ARGs-OAP pipeline (version 2.0) (29). Specifically, 131 metagenomic datasets were searched against the integrated structured ARG database by the Usearch sequence analysis tool with the default parameter (25 aa, *E*-value of 1 × 107, identity of 80%). Potential ARG-like reads obtained from the Usearch results were searched against the integrated structured ARG database [ARDB (Antibiotic Resistance Genes Database), CARD (The Comprehensive Antibiotic Resistance Database), and NCBI-NR] by the Basic Local Alignment Search Tool to ensure accurate annotation. A sequence was annotated as an ARG-like read by meeting the following standards: an E-value cutoff of 1e-7, amino acid similarity of 80%, and minimum alignment length of 25 amino acids. The ARGs-OAP pipeline provided three standardized information including total read number, 16S rRNA gene copy number, and cell number. The abundance of ARG profiles of different sample groups was compared at the type level, the subtype level, and the gene level (the reference sequence level). Results of 16S rRNA gene copy number were selected for the following analysis.

### Antibiotic resistance mechanisms analysis

The CARD database (30) (http://arpcard.mcmaster.ca) was download to classified ARGs detected in the 131 NHPs gut microbiota into different mechanism categories. We mapped the detected ARGs were mapped to CARD database to obtain the resistance mechanism of each ARG. Remarkably, the 15 unassigned subtypes were named ‘others' in this study.

### Statistical analysis

Principal Components Analysis (PCA) and heatmap analysis were performed by STAMP v2.0. Histogram and unpaired t-test analysis were performed by GraphPad v7.0. Alpha-diversity indexes were calculated to estimate microbial diversity between *Macaca sp., M. mulatta, M. fascicularis*, and white-faced capuchins groups. Differences in the relative abundance of the gut microbial ARG features were determined by LEfSe (http://huttenhower.sph.harvard.edu/lefse/).

## Results

### The ARG diversity of *Macaca sp.*, white-faced capuchins, *M. mulatta*, and *M. fascicularis*

**Figure 1:**
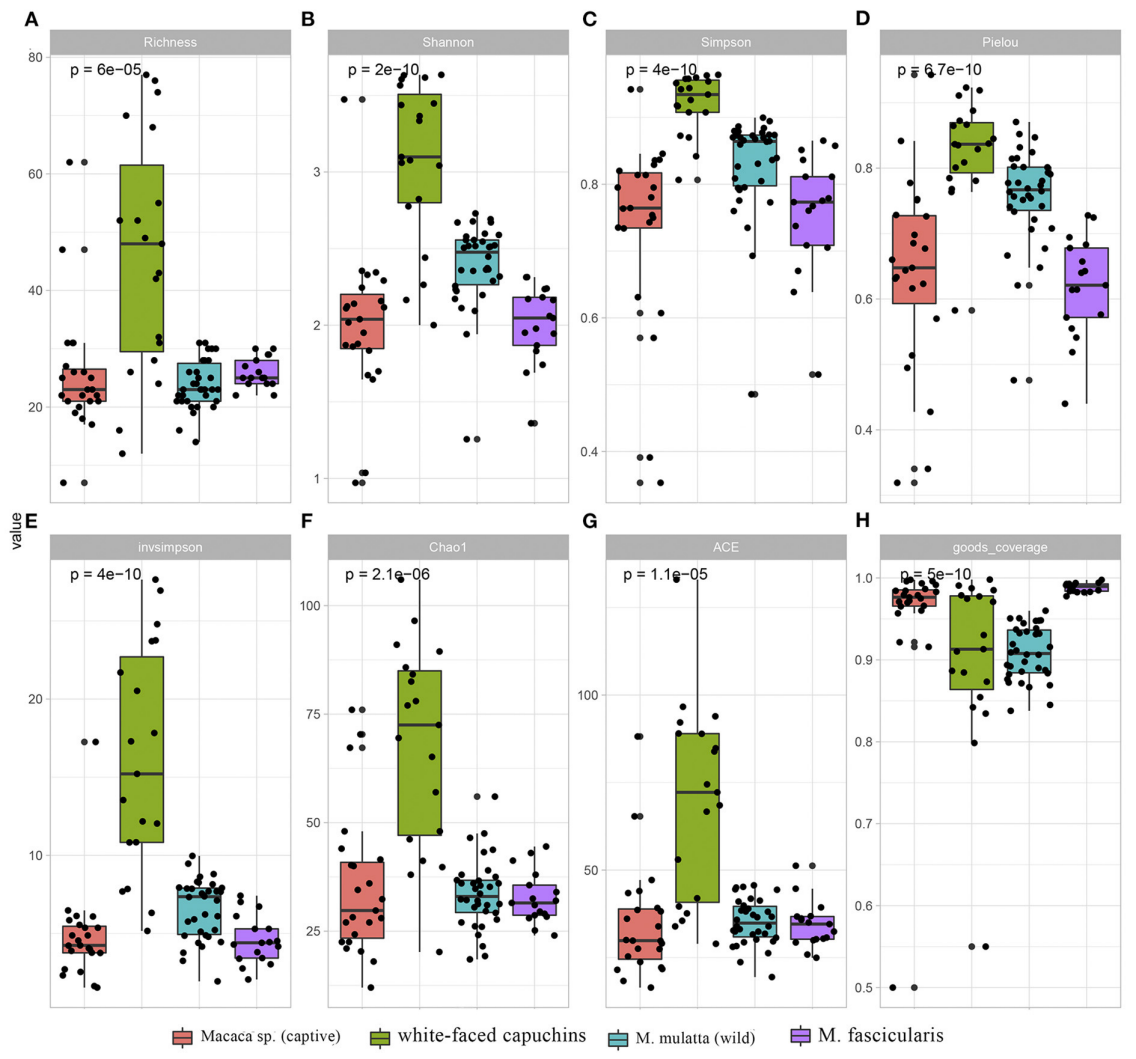
Comparison of ARGs diversity index and richness index across *Macaca sp*. (captive)*, M. mulatta* (wild), white-faced capuchins and *M. fascic*ularis. **(A)** Richness index **(B)** Shanno index **(C)** Simpson index **(D)** Pielou index **(E)** invsimpson index **(F)** Chao1 **(G)** ACE **(H)** goods_coverage.

Except for goods_coverage index, all the other 7 indexes showed that white-faced capuchins had the highest index, followed by the wild *M. mulatta* (Figure 1). *Macaca sp*. in captive and *M. fascicularis* had the lowest index. This indicated that the diversity of ARGs in white-faced capuchins was the highest (Figures 1B,C). However, the goods_coverage indexes showed that *M. fascicularis* and *Macaca sp*. in captive had the highest index, and white-faced capuchins and *M. mulatta* in wild had a relatively low index (Figures 1D,E). The Shannon index, Simpson index, Pielou index, Chao index and ACE index of the *M. mulatta* were higher than the captive *Macaca sp*. (Figures 1B–D,F,G).

### The ARG profiles of white-faced capuchins, *Macaca sp., M. fascicularis*, and *M. mulatta*

**Figure 2:**
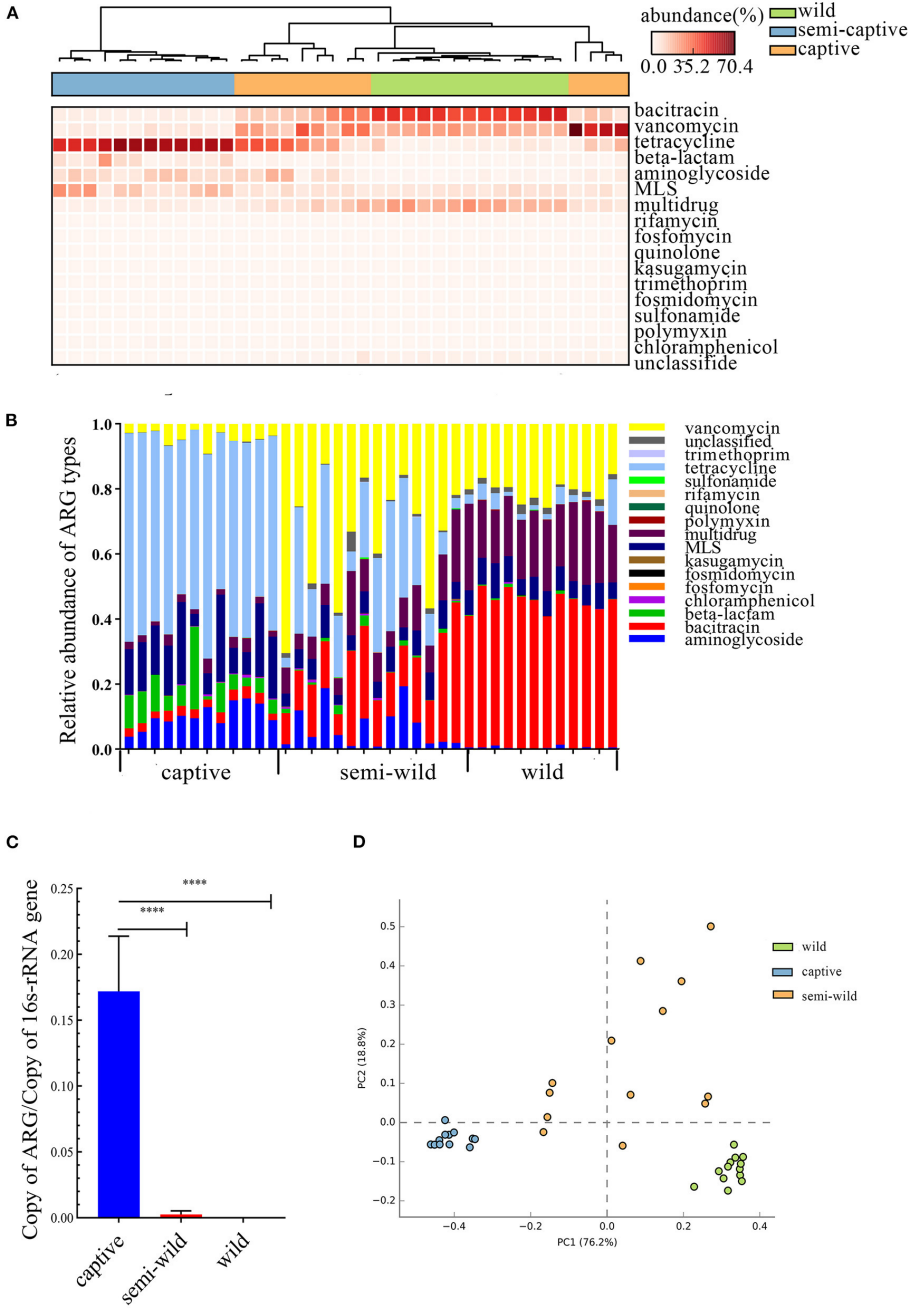
Comparison of *R. roxellana* gut microbiota ARGs between captive, semi-wild, wild. **(A)** Clustering heat map of antibiotics with high relative abundance in 38 samples. **(B)** Relative abundance of ARG types of *R. roxellana*. **(C)** Barplot of total ARG relative abundance of 38 *R. roxellana* (*t*-test, ****P < 0.0001). **(D)** PCA plot of ARG relative abundance of 38 *R. roxellana*.

**Figure 3:**
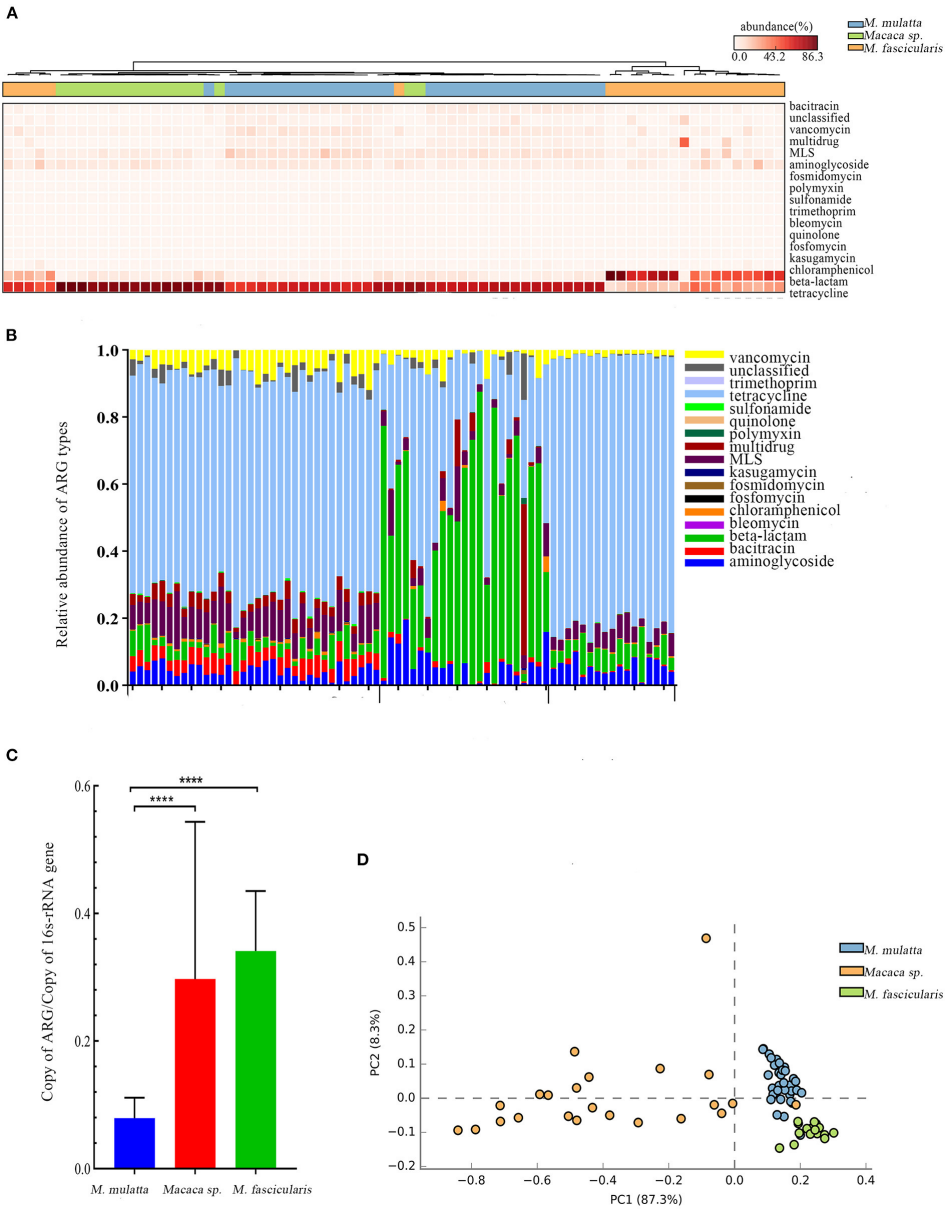
Comparison of Macaca spp. gut microbiota ARGs from three regions. **(A)** Heat map clustering 17 antibiotics of 74 samples. **(B)** Relative abundance of 17 antibiotics of 74 samples. **(C)** Barplot of total ARG relative abundance of 74 samples (*t*-test, ****P < 0.0001). **(D)** PCA plot of ARG relative abundance of 74 samples.

**Figure 4:**
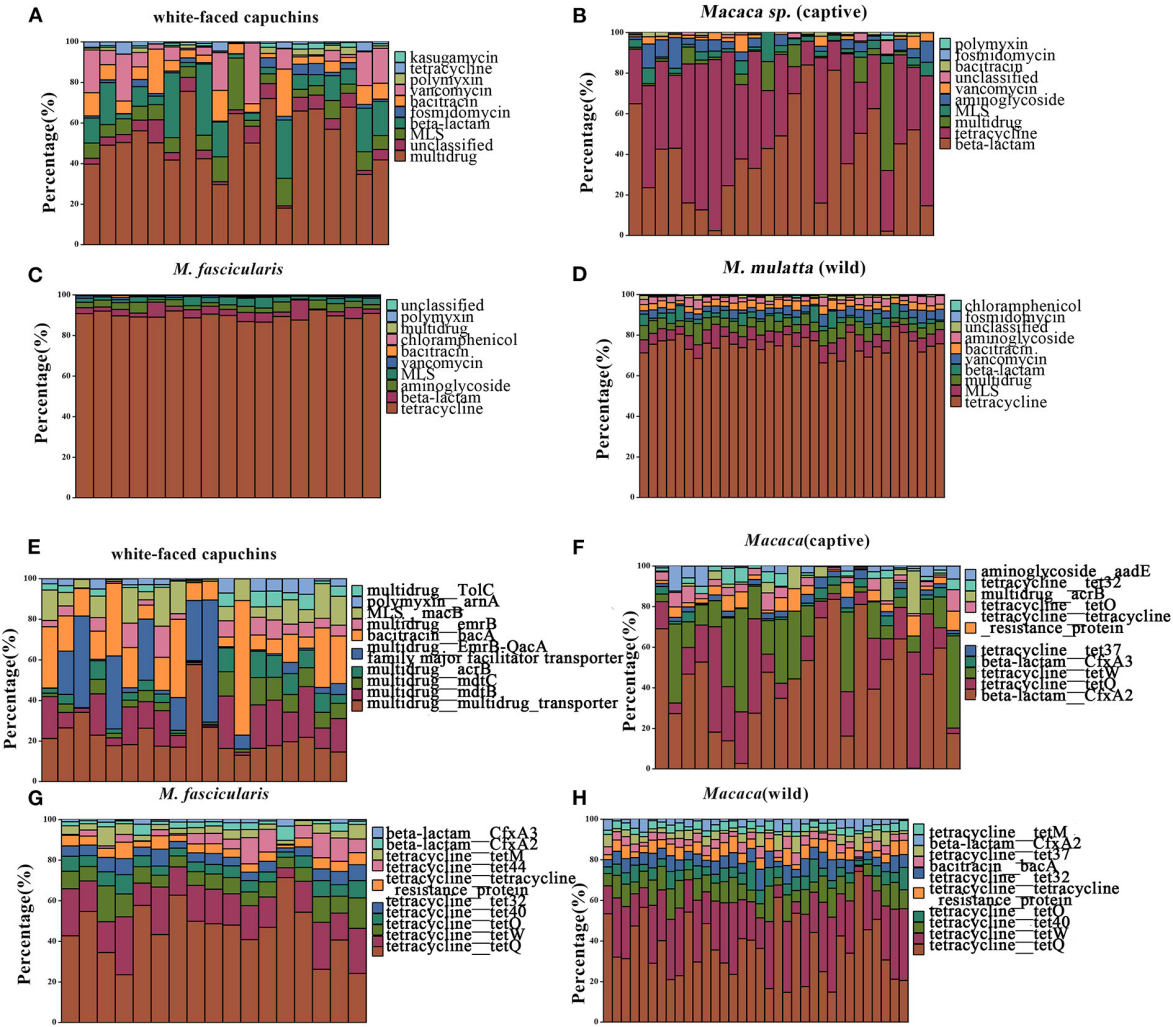
Relative abundances of top 10 type and subtype ARGs. **(A)** The top 10 antibiotic resistance class of white-faced capuchins. **(B)** The top 10 antibiotic resistance class of *Macaca* sp. (captive). **(C)** The top 10 antibiotic resistance class of *M. fascicularis*. **(D)** The top 10 antibiotic resistance class of *M. mulatta* (wild). **(E)** The top 10 antibiotic resistance gene of white-faced capuchins. **(F)** The top 10 antibiotic resistance gene of *Macaca* sp. **(G)** The top 10 antibiotic resistance gene of *M. fascicularis*. **(H)** The top 10 antibiotic resistance gene of *M. mulatta* (wild).

In the study, 19 ARG types were annotated from 131 samples by using the ARGs-OAP pipeline. Seventeen ARG types were identified in white-faced capuchins and *R. roxellana* (Supplementary Figure S1, Figures 2A,B). Sixteen ARG types were found in the wild *M. mulatta* and 12 ARG types were annotated in the captive *Macaca sp*. (Figure 3B). *M. fascicularis* was resistant to 15 ARG types (Figure 3B). The top 10 antibiotic resistance types of white-faced capuchins, *Macaca sp., M. fascicularis*, and *M. mulatta* were presented in Figures 4A–D, respectively. Tetracycline resistance genes had the highest relative abundance in 131 samples, followed by macrolides-lincosamids-streptogramins (MLS), multidrug, and beta-lactam. At the subtype level, 325 ARGs were annotated in 131 samples. Among them, 310 subtypes were classified into four mechanism categories based on the CARD database, of which 145 subtypes belonging to antibiotic inactivation, 110 subtypes to antibiotic efflux, 48 subtypes to antibiotic target alteration, and 7 to antibiotic target replacement (Supplementary Table S2). The top 10 ARGs were presented in Figures 4E–H. *Tet*Q had the highest relative abundance in 131 samples, followed by *tet*W, multidrug_transporter, and *Cfx*A2. The major ARGs of the 3 NHPs differed from one another.

### Influence of captivity on *R. roxellana* gut ARGs

Thirty-eight datasets of *R. roxellana* were downloaded from the NCBI SRA database, including captive group, semi-wild group, and wild group. Seventeen ARG types were found in 38 samples, and the relative abundance of the top 10 ARG types were shown by a heat map (Figures 2A,B). The relative abundance of ARGs in the captive group was significantly higher than that in the semi-wild and wild groups (2.063 ± 0.025 vs. 0.002 ± 0.004, *P* < 0.0001, and 2.063 ± 0.025 vs. 0.0007 ± 0.001, *P* < 0.0001), consistent with the result of our datasets (Figure 2C). The relative abundance of ARGs in the captive group was up to 14-fold higher than that in the wild group. In the captive group, tetracycline was the primary ARG, with relative abundance ranging from 0 to 0.145, followed by 0–0.030 in semi-wild and 0–0.007 in wild. In semi-wild and wild *R. roxellana*, vancomycin, bacitracin, and multidrug had high relative abundance (Figures 2A,B). The PCA analysis confirmed that the three lifestyles clustered separately (Figure 2D). The similar result was seen in the heatmap (Supplementary Figure S3). Diversity analysis found that apart from Pielou index, the diversity index of the captive *R. roxellana* and semi-wild were higher than the wild *R. roxellana* (Supplementary Figure S2). Besides, the marker-ARG of captive *R. roxellana* were tetracycline resistance genes, *tet*W, *tet*Q, tetracycline resistance protein, and *tet*O (Supplementary Figures S4, S5).

### Influence of geographic location on *Macaca sp., M. fascicularis*, and *M. mulatta* gut ARGs

*Macaca spp*. from three places were included to explore whether ARGs were associated with geographic factors (the wild *M. mulatta* from Hezhou, Guangxi, the captive *M. fascicularis* from Beijing, China, and the captive *Macaca sp*. from Minneapolis, USA). At last, 17 ARG types were annotated. The dominant antibiotic resistance of *M. mulatta* and *M. fascicularis* in China were tetracycline, which accounted for 65.71% and 81.27%, respectively. The primary ARG abundance in captive *Macaca sp*. from USA was beta-lactam, accounting for 56.99% (Figures 3A,B). The total relative abundance of ARGs in captive *Macaca spp*. was significantly higher than the wild (unpaired *t*-test, *P* < 0.0001) (Figure 3C), consistent with the result above. PCA analysis was used to evaluate the similarities of the compositions of ARG types in the three groups of *Macaca spp*. (Figure 3D). The samples from the same place clustered more closely, and *M. fascicularis* from China and *Macaca sp*. from USA formed different clusters. Additionally, the captive *Macaca sp*. from the USA was different from those in China, reflecting that the diversity of ARG type was also related to host geography.

### Identification of discriminative ARG relative to captivity and geography

**Figure 5:**
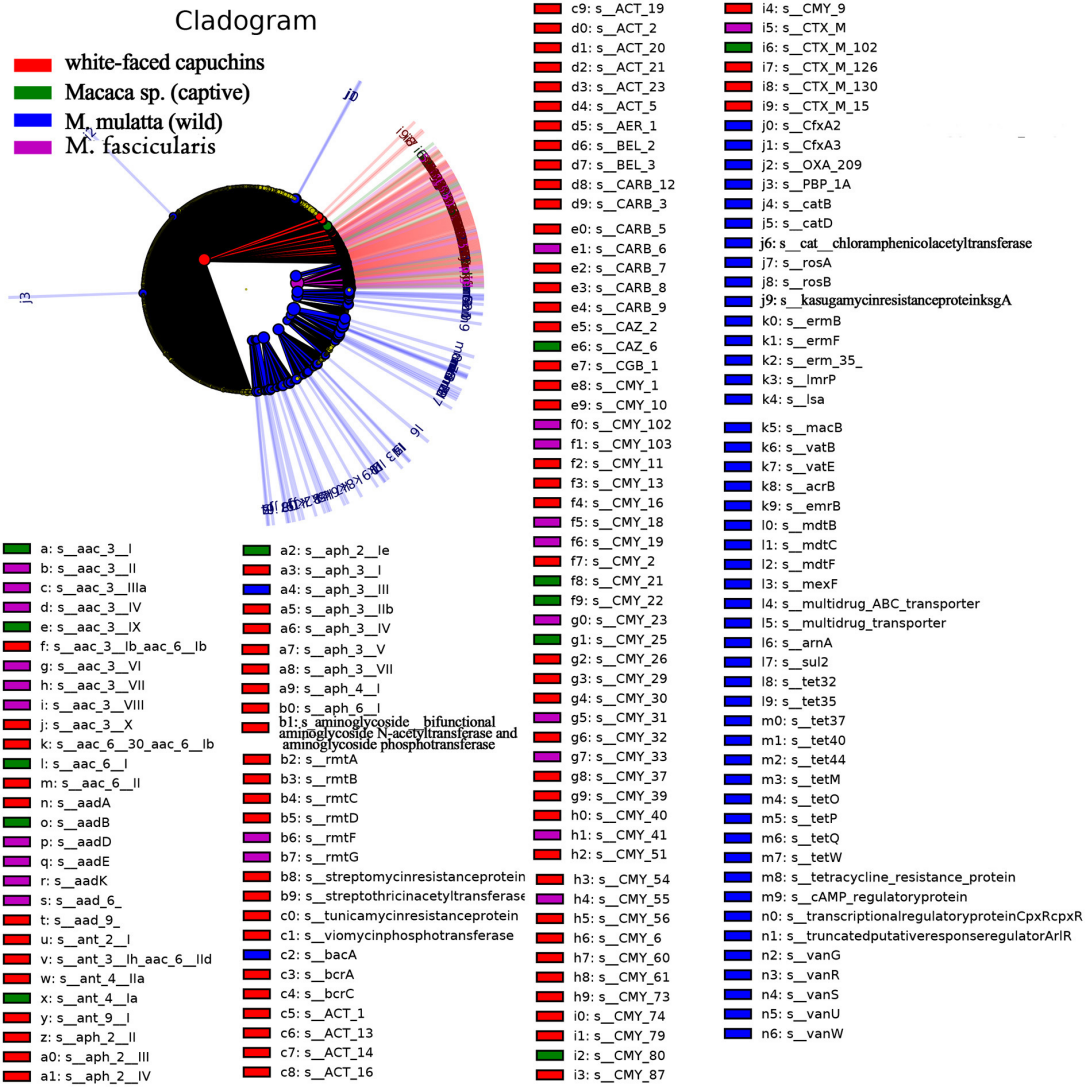
Cladogram of the significantly different ARGs between white-faced capuchins, *Macaca sp*. (captive), *M. mulatta* (wild) and *M. fascicularis* samples. The inner circles are at the ARG type level, while the outer circles are at the subtype level.

**Figure 6:**
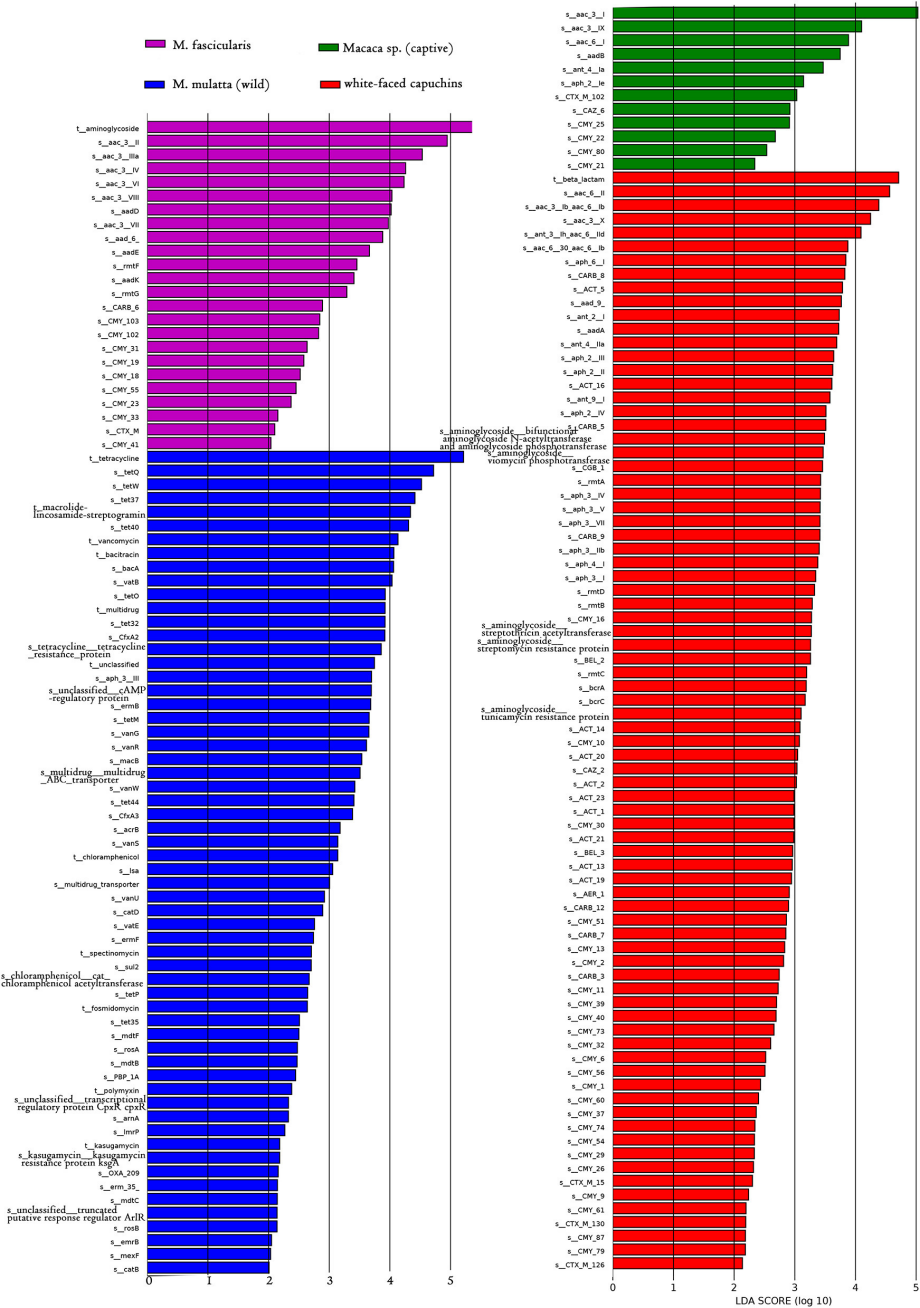
LDA score distribution of the discriminative ARGs between white-faced capuchins, *Macaca sp*. (captive), *M. mulatta* (wild) and *M. fascicularis* fecal microbiomes determined by LEfSe analysis. The red rectangle, green rectangle, blue rectangle, purple rectangle represent white-faced capuchins, *Macaca sp*. (captive), *M. fascicularis* samples, and *M. mulatta* (wild), respectively, of which ARGs' LDA score are more than 2.

LEfSe is an algorithm for high-dimensional biomarker discovery and explanation that identifies genomic features (genes, pathways, or taxa) characterizing the differences between two or more biological conditions (or classes). Thus, this tool enables the characterization of specific ARG profiles and identifies ARG biomarkers in different gut microbial communities. These results showed that the key ARGs in *M. fascicularis* were aac-3-II, aac(3)-IIIa, aac(3)-IV, and aac(3)-VI, which belong to the aminoglycoside resistance gene. The key ARGs in white-faced capuchins were aac-6-II, aac-6-Ib, aac-3-X, and ant-3-Ih-aac-6-IId, which also belong to the aminoglycoside resistance gene. Aminoglycoside resistance genes aac(3)-I, aac(3)-IX, and aac(6')-I were enriched in captive *Macaca sp*. whereas tetracycline resistance genes *tet*Q, *tet*W, and *tet*37 were enriched in wild *M. mulatta* (Figures 5, 6).

## Discussion

Non-human primates are closely genetically related to humans and share many cognitive, physiological and morphological similarities with humans (31). In this study, a total of 131 NHPs were selected, including *Macaca sp., M. mulatta, M. fascicularis*, white-faced capuchins, and *R. roxellana*, to study the impact of captivity and geographic location on the distribution of the NHPs gut resistome.

Researches noted that there were 19 related resistance antibiotics and 325 ARGs in the fecal microbiota of 131 NHPs. Study reveals that both the *M. mulatta* in captive and wild were resistant to tetracycline and with higher abundance compared with other groups. The major resistance of *M. fascicularis* was tetracycline, too. Tremendous tetracycline resistance genes were found in the above three kinds of NHPs, liking *tet*Q, *tet*W, *tet*O, and *tet*40. Yang et al. (32) uncovered that the most abundant ARGs of the gut microbiota of the *M. mulatta* were tetracycline resistance genes and verified by isolation and culture experiments (32). This was also consistent with this study. Aminoglycosides, tetracyclines, and macrolides are the most widely used antibiotics in veterinary medicine worldwide for animal growth promotion and disease control (33, 34). Especially, tetracyclines are broad-spectrum antibiotics and popular first-line antibiotics in human infectious disease treatment (25). The habitat of *M. mulatta* Gupo Mountain in Hezhou is a tourist attraction that produces a lot of human activities. Although tetracycline antibiotics were not used in the wild *M. mulatta*, long-term human activities and widespread use of tetracycline antibiotics in other species have caused an extensive global tetracycline resistance (31, 35, 36). It may be explained the prevalence of tetracycline resistance genes in NPHs. Besides, this study found that 145 subtypes belong to antibiotic inactivation. Similar results have been reported that antibiotic inactivation was the dominant resistance mechanism in swine and humans (37).

The effect of captivity on intestinal microbial resistance in animals is still unclear. Previous researches show that captivity influences the gut resistome structure by altering the gut microbiota composition. Thus, comparison with wild animals, captive breeding may affect the diversity/abundance of resistant genes by affecting their own intestinal microbial structure in a variety of ways, such as dietary changes, drug treatment, and reducing contact with other individuals, species, and variable environmental substrates as a source of bacterial diversity (16, 38, 39). Previous research also reveals that semi-captive wildlife might harbor a higher diversity of antimicrobial-resistant genes (40). Research on captive and wild baboon gut microbiota and their resistome found that exposure to humans is associated with changes in gut microbiota composition and resistome expansion compared (15). Rolland et al. (41) found that compared with baboons that do not contact humans, baboons that directly contact humans can detect a higher level of antibiotic resistance (41). In these results, both white-faced capuchinsand *M. mulatta* in wild had high diversity index. Further analysis of *R. roxellana* found that captive *R. roxellana* had significantly higher diversity and abundance than wild and semi-wild. Therefore, diversity index analysis can not tell the direct relationship between captive and wild NHPs. It is certain that lifestyle is an important factor that influences the ARGs diversity of NHPs. Besides, a comparison of *M. mulatta* in China and the USA revealed that ARG was also influenced by geography but to a lesser extent. Consequently, a concluded that gut resistome of NHPs were more substantially affected by the captive environment than geography or host.

Compared with the gut microbiota ARGs of *M. mulatta* and *M. fascicularis*, the diversity of gut microbiota ARGs of white-faced capuchins was the highest. Various ARGs of white-faced capuchins that were significantly different from *M. mulatta* and *M. fascicularis* in this study, indicating that the ARGs may have spread to the habitats of white-faced capuchins. Strangely, tetracycline resistance genes *tet*Q, *tet*W and *tet*37 were found in the wild gut microbiota of *M. mulatta*. The spread of ARGs in wild NHPs is more worrisome because it may lead to a large-scale environment polluted that increasingly animal antibiotic-resistant infections cannot be treated including endangered animals. Research showed that the frequent use of antibiotics has led to antibiotic selection pressure causing the prevalence of antibiotic-resistant bacteria (42). Therefore, antibiotics must be used reasonably to reduce the selection pressure. Measures should be taken to reduce resistance development and spreading of resistant bacteria. Under the one-health approach, a holistic perspective on antibiotic resistance, including humans, animals, and the external environment needs to take on Bengtsson-Palme et al. (43). Furthermore, models should be built to demonstrate and predict how resistance emerges and disseminates by increasing knowledge of influence factors and the mechanism of transmission of drive resistance. All in all, these results provide references for NHPs' gut resistome studies.

## Conclusion

The study described a comprehensive *R. roxellana, M. mulatta, Macaca sp*. *M. fascicularis*, and white-faced capuchins gut resistance gene catalog and revealed the abundance and diversity of ARGs of them. The total ARGs relative abundance in the captive animal was higher than in the wild and tetracycline was the predominant resistance in both captive and wild NHPs. Captivity was considered to be the primary influence factor of host gut microbiota ARGs abundance and diversity. This study helps to improve the knowledge and understanding of NHPs' gut flora ARGs and strengthen the management of antibiotics and the prevention of human diseases.

## Data availability statement

Publicly available datasets were analyzed in this study. The names of the repository/repositories and accession number(s) can be found in the article/Supplementary material.

## Author contributions

HH designed and constructed the manuscript, downloaded and analyzed of the datasets, generated the major figures and tables, and completed the writting.

## Conflict of interest

The author declares that the research was conducted in the absence of any commercial or financial relationships that could be construed as a potential conflict of interest.

## Publisher's note

All claims expressed in this article are solely those of the authors and do not necessarily represent those of their affiliated organizations, or those of the publisher, the editors and the reviewers. Any product that may be evaluated in this article, or claim that may be made by its manufacturer, is not guaranteed or endorsed by the publisher.
