# Supplementary material for: Captivity and geography influence the antibiotic resistome of non-human primates
Source: Front Vet Sci. 2022 Nov 18;9:1020276. doi: 10.3389/fvets.2022.1020276 (PMC9716204; doi:10.3389/fvets.2022.1020276)
Supplement: Supplementary file 1 [file Data_Sheet_1.docx]

**Supporting information for Captivity and Geography Influence the Antibiotic Resistome of non-human primates**

Hongli Huang^12*^

^1^Clinical Biological Specimen Bank, Discipline Construction Office, The First Affiliated Hospital of Guangxi Medical University, Nanning, Guangxi, 530021, China.

^2^ Life Sciences Institute, Guangxi Medical University, Nanning, Guangxi, 530021, China.

Corresponding Author:

Hongli Huang

No 6 Shuangyong Road, Nanning, Guangxi 530021, China.

Email address:15296536692@163.com


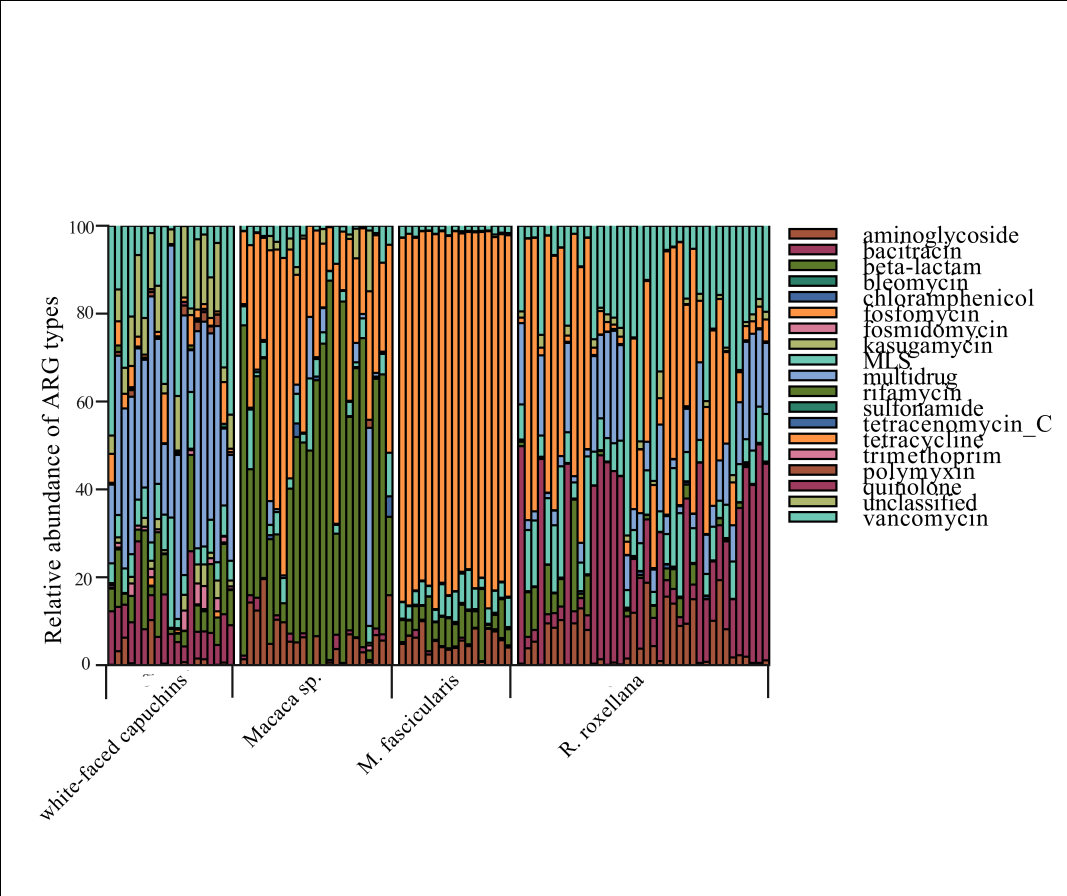


Figure S1 The ARG types relative abundance of white-faced capuchins, *Macaca sp.*, *M. fascicularis,* and *M. mulatta*


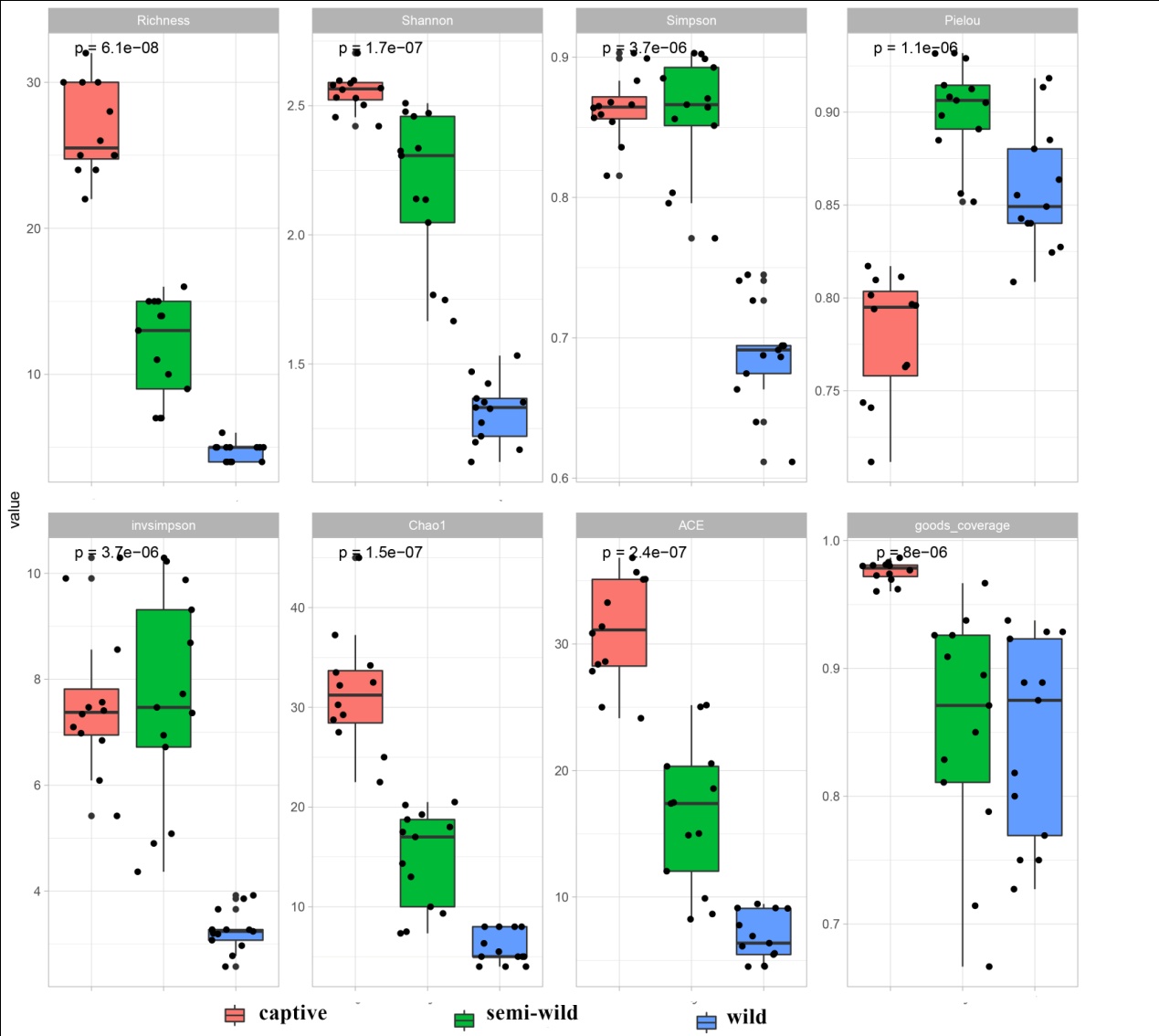


Figure S2 Diversity index analysis of *R. roxellana* antibiotic resistance genes.


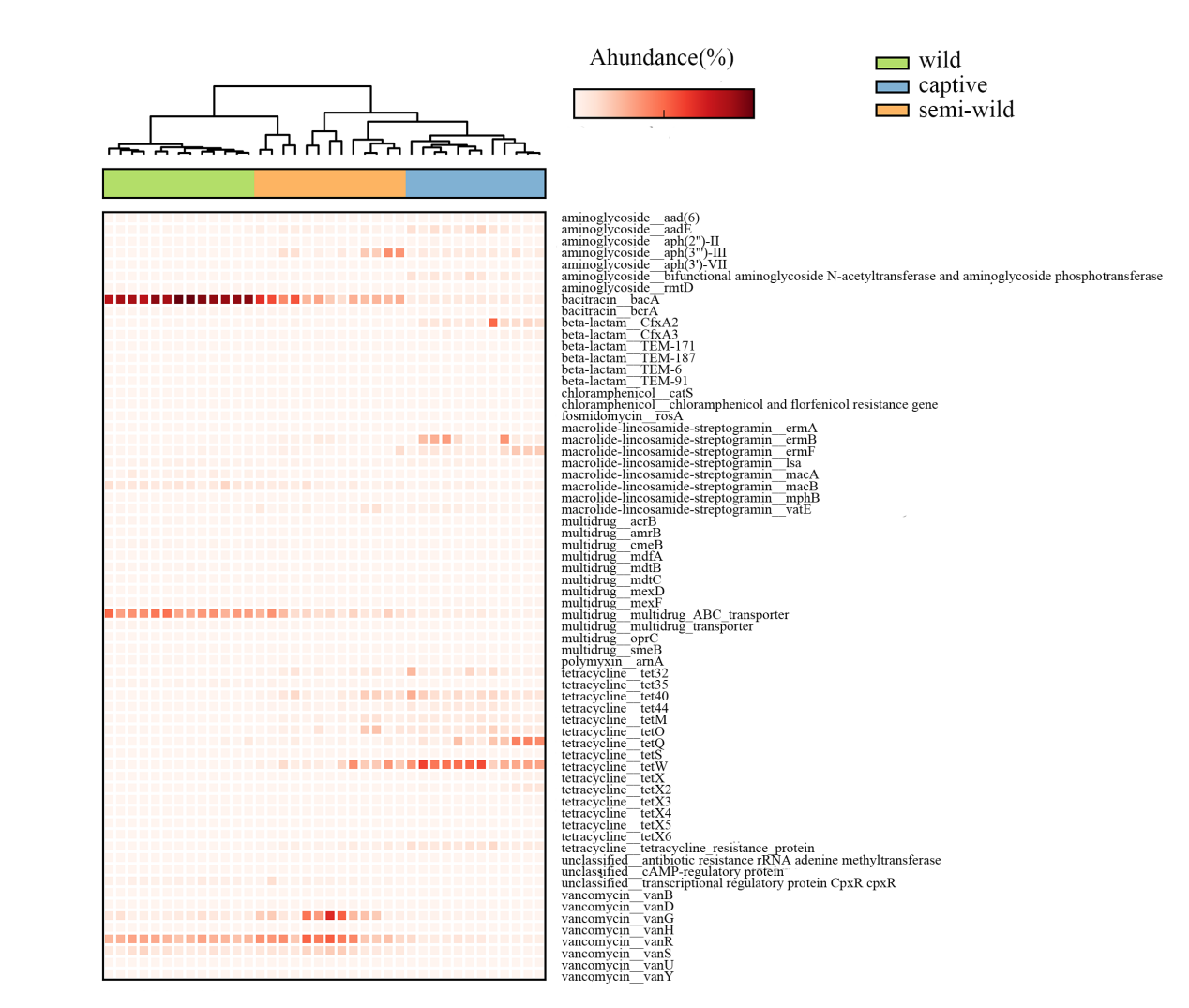


Figure S3 A cluster heatmap of the gut microbiota antibiotic resistance genes of *R. roxellana*.


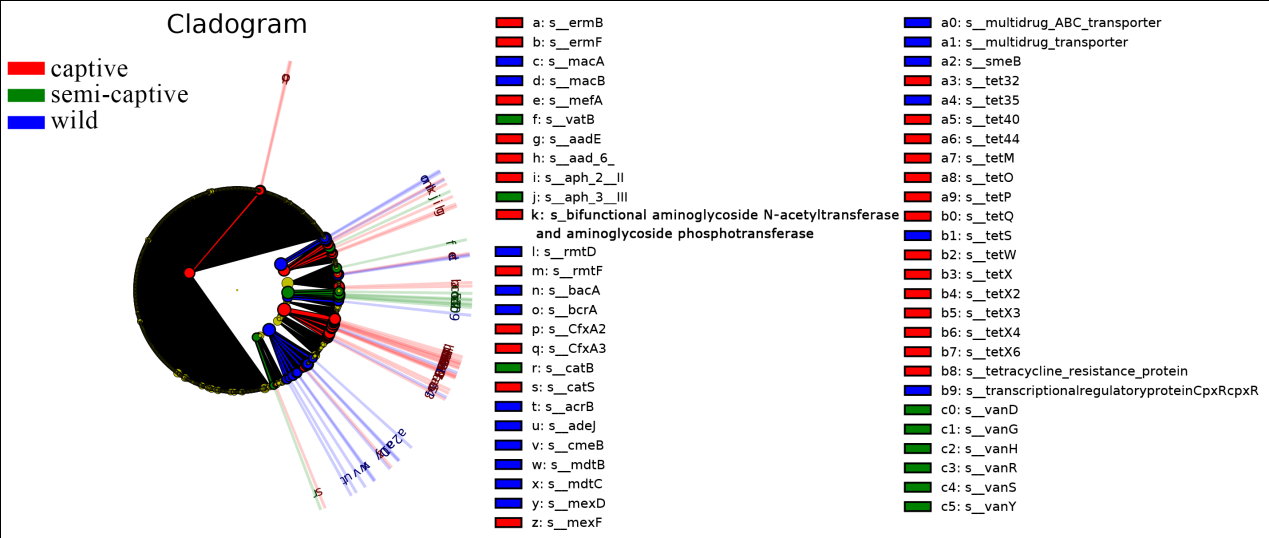


Figure S4 Cladogram of the significantly different gut microbiota ARGs of *R. roxellana* by LEfSe analysis.

.


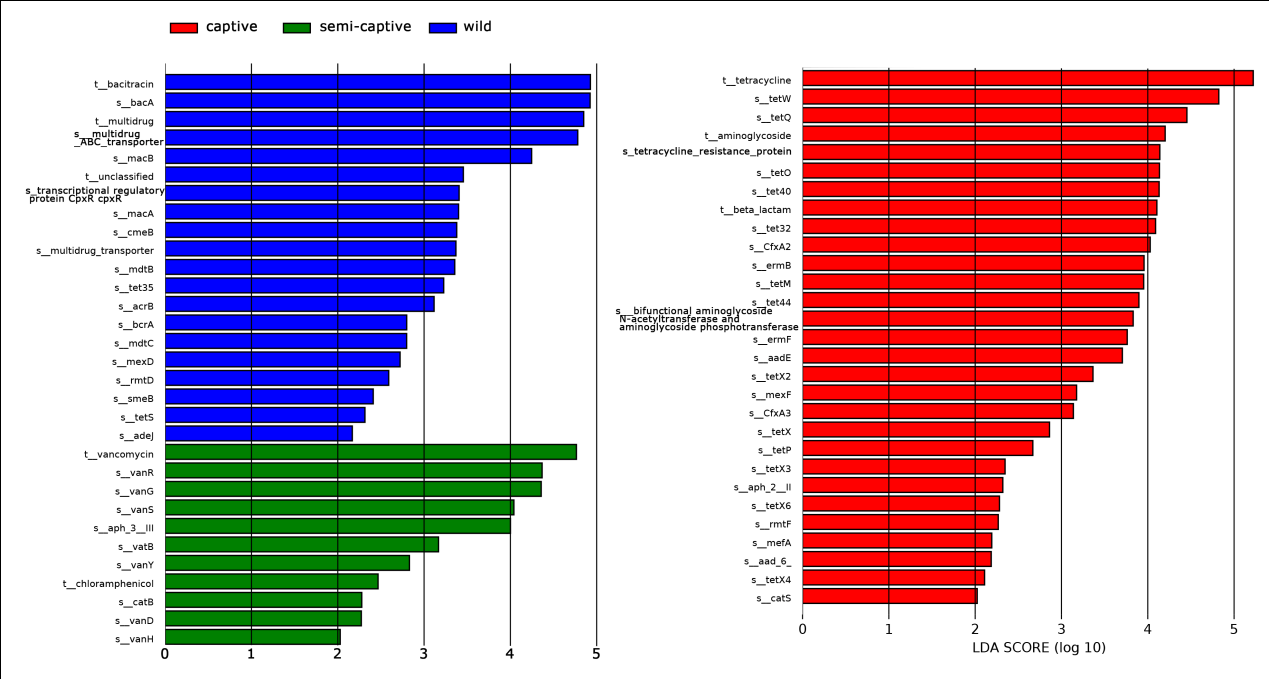


Figure S5 LDA score distribution of the discriminative gut microbiota ARGs of *R. roxellana* by LEfSe analysis.
